# *Salmonella enterica* subspecies *enterica* serovar Choleraesuis in a Swedish gilt-producing herd, a case report

**DOI:** 10.1186/s40813-023-00329-7

**Published:** 2023-07-28

**Authors:** Johanna Fjelkner, Cecilia Hultén, Magdalena Jacobson, Erik Nörregård, Beth Young

**Affiliations:** 1 Farm & Animal Health, Box 164, Staffanstorp, 245 22 Sweden; 2 National Veterinary Institute, Uppsala, Sweden; 3 Swedish University of Agricultural Sciences, Uppsala, Sweden

**Keywords:** *Salmonella*, Choleraesuis, Salmonellosis, Pigs, Elimination

## Abstract

**Background:**

When *Salmonella enterica* subspecies *enterica* serovar Choleraesuis (*S.* Choleraesuis) was detected in faecal samples collected within the Swedish *Salmonella* surveillance program from a gilt multiplying herd in September 2020, *S.* Choleraesuis had not been detected in domestic pigs or wild boar in Sweden for over 40 years. This report describes the subsequent investigation, identification of possible entry routes and measures undertaken to eliminate the pathogen from the herd.

**Case presentation:**

In accordance with Swedish regulations, pig movements to and from the farm were restricted, internal biosecurity measures were enhanced, and a test-and-remove strategy was implemented. Testing included repeated faecal sampling, tissue samplings from all dead or euthanized pigs, and serological sampling of replacement gilts. Epidemiological investigations included scrutinising of production records, employee interviews, analysing feed and environmental samples, faecal samples from the herd’s purebred gilt supplier, and tissue and faecal samples from wild boars in the adjacent area. Testing of in-contact herds receiving gilts (n = 15) or 30-kg pigs (n = 7) from the multiplier included whole-herd faecal sampling and tissue cultures from pigs that died with signs of septicaemia. In total, *S.* Choleraesuis was detected in 12/4200 faecal and 5/1350 tissue samples from the herd, and the corresponding groups of pigs were euthanized. All feed and environmental samples as well as samples from the gilt supplier were negative. Testing of contact herds resulted in the identification and culling of one group of *S*. Choleraesuis-positive gilts. Replacement gilts introduced to the herd from January until May 2021 remained serologically negative during a surveillance-period of five months.

**Conclusion:**

Although speculative, the epidemiological investigation identified indirect transmission from wild boar as possible source of introduction to the herd. Whole-genome sequencing of *S*. Choleraesuis isolates from wild boar in the area showed that they clustered with isolates from the herd. Repeated testing of the herd indicated that the test-and-remove strategy was successful. In August 2021, all restrictions were removed, and the herd was re-instated as a gilt producing herd. Compensation from the Swedish state to the farmer for production losses, culled animals and extra costs associated with the elimination cost totalled SEK 7 992 234.

## Background

To date, more than 2400 *Salmonella* serovars have been identified, most of which have a broad host range. Several serovars are adapted to a specific host including *Salmonella enterica* subspecies *enterica* serovar Choleraesuis (*S.* Choleraesuis) which is host-adapted to pigs. It may cause septicaemia and enterocolitis, and, particularly in weaned pigs less than five months of age, the case fatality rate is often considered to be high [1]. Although rare, the serovar can also produce severe disease in other species, including humans [2]. In the past, *S*. Choleraesuis was one of the most common serovars isolated from pigs worldwide and it is still a common finding in pigs in North America and Asia [3, 4]. However, an investigation undertaken in 2008–2009 found that *S.* Choleraesuis was a relatively rare serovar in the domestic pig population within the EU [5, 6]. In Europe, outbreaks of *S.* Choleraesuis have been reported in Denmark in 1999–2000 (one herd) [7] and in 2012–2013 (four herds) [8]. *S.* Choleraesuis has not been detected in domestic pigs in Sweden for over 40 years [9].

A national *Salmonella* control program that covers the entire chain from feed to food has been in place in Sweden since the 1950s [9], aimed at ensuring all Swedish food of animal origin is free from *Salmonella*. This control program is governed by the Swedish Act on Zoonoses (SFS 1999:658) and its regulations, and it has previously been described by others [10]. As part of the program, all gilt-producing pig herds must be tested annually by faecal culture [9]. If *Salmonella* is confirmed, the herd is put under restrictions and all animal movements to and from the herd are stopped. An epidemiological investigation is performed, and a plan to eliminate *Salmonella* from the herd is made. The farmer is compensated by the Board of Agriculture for production losses, extra workload, cleaning and disinfection costs, and culled animals. The Board of Agriculture also covers the costs for an appointed veterinarian and diagnostic testing [10].

The aim of this case report is to describe the unexpected finding of *S.* Choleraesuis in a Swedish gilt-producing pig herd, identified through the *Salmonella* surveillance program, and the measures undertaken to attempt to eliminate the pathogen from the herd.

## Case presentation

In September 2020, faecal samples were collected from the case herd as a part of the compulsory annual *Salmonella* surveillance program for gilt-producing herds [9]. Samples were collected from sows in each of the two farrowing rooms on the farm. In total, 50 sows were individually sampled by taking faecal samples from the pen floor. The samples were pooled by five for a total of ten pooled samples which were sent to the National Veterinary Institute for *Salmonella* analysis by culture in accordance with EN ISO 6579-1:2017 [11]. Four of the ten pools tested positive for *Salmonella* spp. All four samples originated from the same farrowing room. The herd was immediately put under restrictions by the Swedish Board of Agriculture and pig movements to and from the farm were prohibited.

### Farm description

The 220-sow herd produced Topigs Norsvin 70 (TN70) hybrid gilts for breeding. The herd purchased seven-month-old Norwegian Landrace maternal breeding-line replacement gilts from a single Swedish nucleus herd. All breeding was done by artificial insemination, using fresh semen purchased from a Swedish boar stud.

The herd employed a three-week interval batch-farrowing system. Each batch consisted of a group of 32 sows and their offspring, with a total of seven groups (referred to as Group 1 to Group 7). All sows in a batch farrowed in one farrowing room in individual pens with a combination of solid/slatted flooring and straw bedding. Cross-fostering was used at birth to equalise litter size. The piglets were weaned at five weeks of age.

At weaning, sows were moved to the insemination room where they were held batch-wise in pens with deep straw bedding and feeding stalls. After five weeks of gestation, the batch was moved to another, similar, pen within the same room. After eight weeks of gestation, the sows were moved to a gestation unit in a separate building on the same site, where they stayed until farrowing. The sows were moved between the two buildings in a transport wagon and did not have any direct contact with the outdoor environment.

At weaning, all piglets from a batch were moved to one of the three nursery rooms at the farm, where they were sorted into pens by size. Piglets that fell behind were moved to a separate pen approximately two weeks after weaning. The piglets remained in the nursery for eight weeks.

The farm sold replacement stock to 15 different Swedish piglet-producing herds. Four different categories of gilts were sold: 30-kg gilts, five-month-old, seven-month-old, and pregnant gilts. All castrates and excess gilts were sold at approximately 30 kg on the open market to different finisher farms. Gilts that were not sold at 30 kg were moved from the nursery to one of six grower rooms. After 18–19 weeks, gilts that were to be sold as seven-month-olds or as pregnant gilts were moved to the insemination room where they were held separately from the sows in pens of 12–16 animals.

The farm had a non-residue feeding system. They produced their own liquid feed for sows, growers and gilts using home-grown grain (a mix of barley, triticale and mixed grain bought at the open market) and a heat-treated concentrate that was mixed with water, soy, and Distillers dried grain with solubles (DDGS). The suckling piglets were fed a heat-treated commercial creep feed from approximately ten days of age. For the first ten days after weaning, the piglets were fed a commercial feed mixed with water. Thereafter, the feed was gradually switched to the farmer’s own mix. Peat (not heat-treated) was used as bedding material for the first week after weaning, thereafter being replaced by straw. The peat was delivered to the farm in plastic sacks and stored in a barn. Straw was stored outdoors immediately after harvest and thereafter in three separate barns.

All sows were vaccinated against *Escherichia coli*, porcine parvovirus and *Erysipelothrix rhusiopathiae*. Piglets were vaccinated against porcine circovirus type 2 (PCV2) and *Mycoplasma hyopneumoniae*. The herd was free from porcine reproductive and respiratory syndrome virus (PRRSV), *Brachyspira hyodysenteriae, Sarcoptes scabiei* var *suis* and toxin-producing *Pasteurella multocida.* The farms’ pre-existing biosecurity measures are described in Table 1.

**Table 1:** Pre-existing on-farm biosecurity measures before *Salmonella* was detected in a Swedish gilt-producing herd

Logbook of all visitors
Shower in - shower out with complete change of clothes for both staff and visitors
No outdoor access for pigs
Separate boots for use when walking outdoors between the two different barns
Professional rodent control with traps and poison, no bird access to barns (netting in of windows and ventilation)
Three-week isolation for replacement stock
Separate manure scrapers for each room
Cleaning with high-pressure washer and disinfection between each batch in farrowing rooms, nursery rooms and grower units.
Cleaning once a year with high-pressure washer in insemination and gestation room.
Transport wagon to move pigs between barns.
Loading system with a clear boundary between the farm and the truck driver
A plan for deadstock management with on-farm incineration

### Outbreak management

#### Initial testing and culling

To investigate the extent of faecal shedding of *Salmonella* spp., faecal samples were collected from all pigs in the herd. Sows in the farrowing rooms were individually sampled and pooled pen samples were collected from the floor in the nursery and grower rooms. Sows and gilts in the insemination and gestation rooms were sampled using pooled faeces from the straw bedding, with one pool per every ten animals in a pen. All pooled samples were collected by selecting faeces from different areas in the pen to ensure that as many animals as possible were represented in the sample.

Three individually sampled sows from one of the farrowing rooms (Group 2) tested positive for *Salmonella* spp. In addition, three pens in one nursery room tested positive. The positive nursery pigs had been weaned one week earlier and were the offspring of sows in Group 1. The remaining samples were negative. The *Salmonella* spp. was identified by whole-genome sequencing as *Salmonella enterica* serovar Choleraesuis.

As a result, of these findings, all sows and piglets belonging to Group 2, and nursery pigs from sows in Group 1, were culled. Sows from Group 1 were not culled as all pooled samples from the group were negative. At culling, the animals were removed via the backdoors of the rooms to avoid potential contamination of the internal corridors.

A second sampling with individual rectal samples was performed in the insemination and gestation rooms to ensure that all animals were included (n = 288). All of these samples were negative for *Salmonella* spp., including all individual samples from sows in Group 1. However, on the same day, one gilt was found dead in one of the pens. A post-mortem indicated sepsis and *S.* Choleraesuis was identified through tissue culture. Therefore, all gilts in the insemination room originally intended for sale were culled. Tissue samples from tonsils, mesenteric lymph nodes and colon were collected from 70 of the 120 gilts. Samples from mesenteric lymph nodes and colon, originating from one gilt, were positive for *S.* Choleraesuis.

#### Biosecurity and hygienic measures

To minimize the risk of spread of the bacteria from and within the herd,, both external and internal biosecurity measures were strengthened immediately after the initial test results came back from the laboratory according to Table 2.

**Table 2:** Strengthened biosecurity measures following the detection of *Salmonella* in a Swedish gilt-producing herd

Separate boots for each room
Using gloves when handling pigs and change of gloves between rooms (at minimum)
No cross-fostering
Dry disinfectant used in corridors before and after moving pigs
Minimal mixing of piglets at weaning
No moving of pigs to sick pens
Cleaning of equipment such as castration equipment, wash robot, high pressure water-hoses after use
Cleaning of tractor after replacing deep straw bedding
Rigorous washing protocol after emptying a room.

Extended cleaning measures were undertaken to eliminate *S.* Choleraesuis from the herd. In rooms where *Salmonella* had been detected, all disposable materials such as scrapers, brooms and wooden boards were discarded. All loose inventory such as heat lamps, plastic boards etc. were cleaned separately. The room was thoroughly pre-cleaned using a washing robot. This was followed by application of a detergent (EWA® FOAM ultra (Theseo Deutschland GmbH; Wietmarschen; Germany), a cold-water, high-pressure wash and finally disinfection with a glutaraldehyde-based disinfectant (Aldekol DES® FF; EWABO Chemikalien & C0. KG; Wietmarschen; Germany), after which the room was left to dry for 3–5 days. If low temperatures and/or high humidity prolonged the time needed for drying, extra heat sources were used to dry the rooms more effectively. Repairs to pen floors, walls and roofs and installation of new creep area roofs was performed whenever deemed necessary. All concrete walls were painted. Finally, the pens were disinfected and left to dry once more. After each cleaning, the room was inspected by a veterinarian, and environmental samples were collected and analysed for *Salmonella* spp.. Afterwards, calcium hydroxide (slaked lime) and water was applied to all solid and slatted floors and left to dry before a new batch of pigs entered. The subsequent cleanings of the farrowing, nursery and grower rooms between batches was performed in a similar way.

The insemination room could not be completely emptied of animals and therefore the washing protocol was adjusted. First, following their euthanasia, the pens previously housing gilts intended for sale, were cleaned. The remaining sows were then moved to the clean area, and the other half of the room was cleaned. All cleaning in the insemination room was performed manually with low water-pressure to minimise the risk of aerosol spread of *Salmonella* to the animals remaining in the room. All inventory in the feeding stalls were manually cleaned using a detergent and rinsed with water. From this point, the original protocol was used.

To decrease the risk of environmental contamination and spill-over to wild boars from the farm, measures were taken to decontaminate manure from the herd before spreading it on the fields. The liquid manure was mixed with 2% urea and stored for one week before being spread. The solid manure was placed in stacks covered with calcium hydroxide and composted for at least six months before spreading [12].

**Fig. 1:**
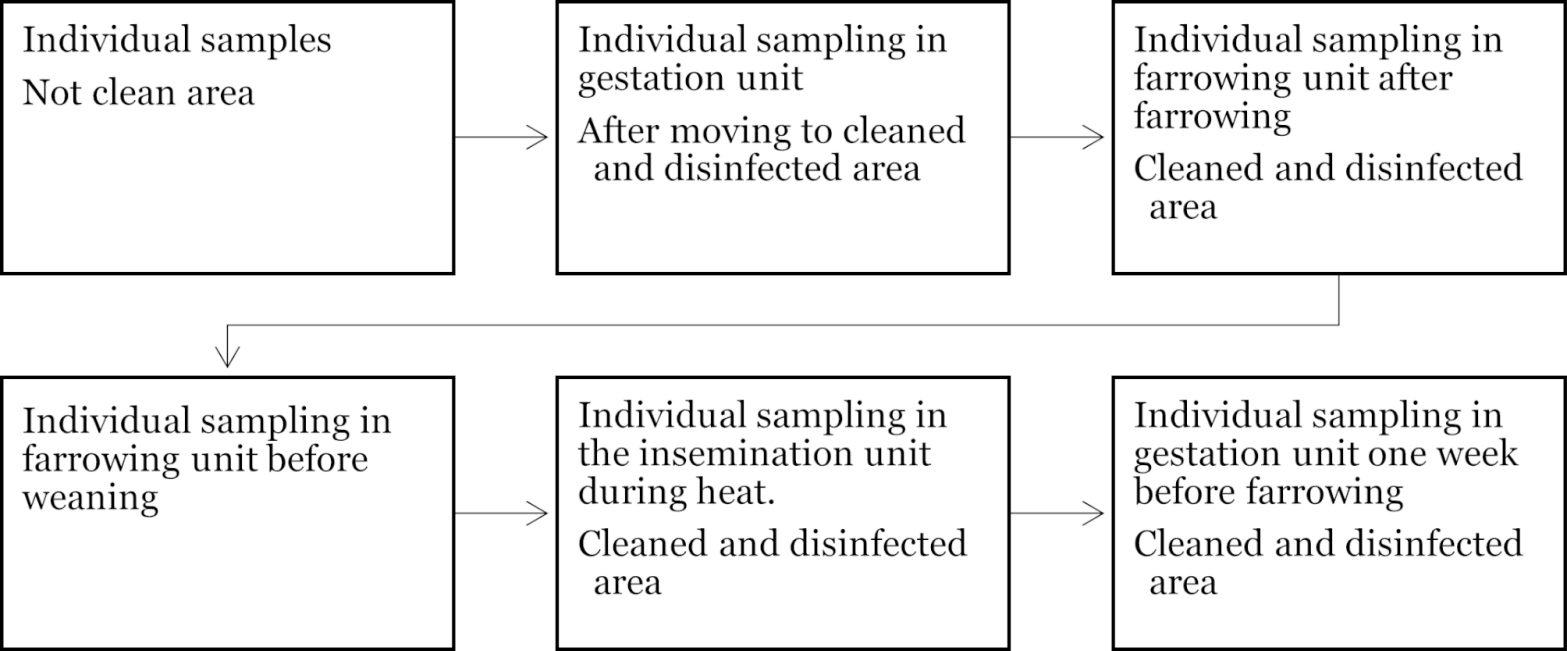
Schematic plan for repeated faecal sampling of sows in a Swedish gilt-producing herd

**Fig. 2:**
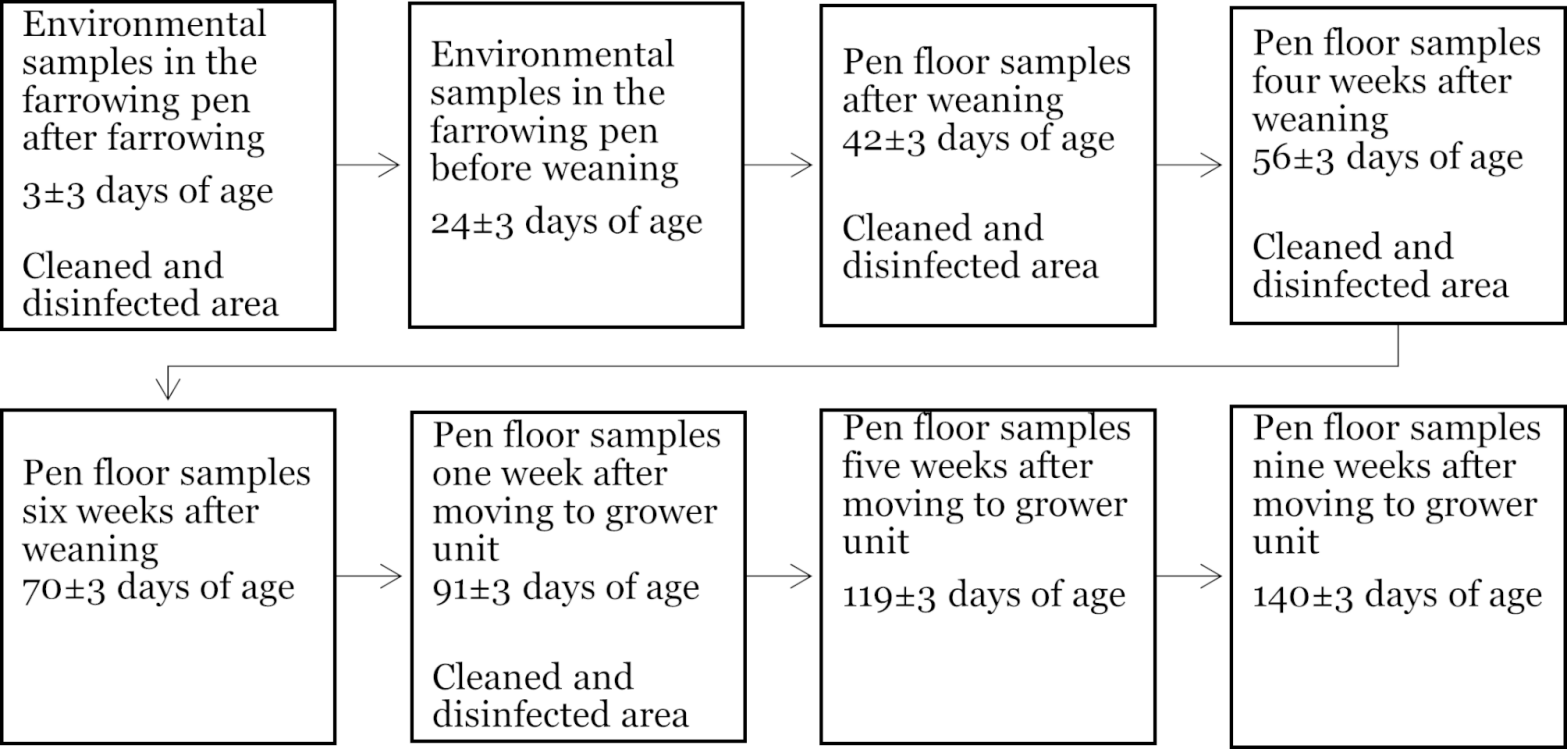
Schematic plan for repeated faecal sampling of growing pigs in a Swedish gilt-producing herd

Further sampling in the herd was planned to confirm the freedom from *Salmonella* spp. following the elimination procedure. The sensitivity of the faecal sampling for *Salmonella* spp. was expected to be low due to the intermittent shedding and non-uniform distribution of the bacteria in faeces [13]. Therefore, a schedule of repeated samplings was planned for both sows and piglets. Each sow was sampled individually five times during one production cycle. Piglets were sampled by pen three times in the nursery and three times in the grower unit. If samples were positive for *Salmonella* the whole group of pigs was culled. The sampling plan is schematically described in Figs. 1 and 2.

In addition to the faecal samples, tissue samples from the tonsils, mesenteric lymph nodes and colon were collected from all pigs older than seven days that died or were culled and sent for analysis. This included all sows selected for culling due to age or inadequate production results after weaning.

Samples were collected according to the schedules described in Figs. 1 and 2 from October 2020 until June 2021. Isolates were further characterised by whole-genome sequencing. In total, 4200 faecal samples were collected and analysed. Twelve of these samples were positive for *S.* Choleraesuis, all within the first two months after first detection of the pathogen. Thereafter, no *Salmonella* spp. was detected in faeces. Five of the 1350 tissue samples collected from dead or euthanized pigs in the herd were positive for *S.* Choleraesuis. The samples were collected from three different individuals. The last positive tissue samples (mesenteric lymph nodes and colon) were detected in March 2021 from a culled sow, who had repeatedly tested negative in faecal samples. This individual had been sampled 6 times, with the first sample taken two weeks after the *S*. Choleraesuis was first isolated in the herd, and the sixth sample collected 10 days prior to culling. The results of the sampling are presented in Table 3. In total, the positive samples resulted in euthanasia of one group of lactating sows with piglets, half a group of pregnant sows and two groups of weaned pigs.

**Table 3:** Schedule over the number of faecal and tissue samples collected each week and the number of *Salmonella*-positive samples in a Swedish gilt producing herd

Week	0	1^1^	2	3–7	8	9–26	27	28	29	30^3^	31–43	44^3^	45–48	49^3^	50^2^
*Salmonella* detected in faecal samples	YES	YES	NO	NO	YES	NO	NO	NO	NO	NO	NO	-	-	-	-
No. of samples	10	582	288	441	106	1449	118	46	67	104	989	-	-	-	-
*Salmonella* detected in tissue samples	NO	NO	YES	NO	NO	NO	YES	NO	NO	NO	NO	-	-	-	-
No. of pigs sampled	-	-	1	100	15	160	14	15	10	15	120	-	-	-	-

^1^: Sanctions implemented; ^2^: Sanctions lifted; ^3^: blood sampling of purchased gilts

Replacement gilts introduced to the farm from January to May 2021 were used as sentinel animals and were serologically tested three times at four-week intervals during late spring and summer. The samples were analysed with a Multiplexed Fluorescent ImmunoAssay (MFIA) for detection of antibodies to *Salmonella* enterica serogroup B and C1 [14]. All gilts were serologically negative in all three samplings.

#### Outcome of outbreak management

During the elimination and sampling period from October 2020 until June 2021, no animals were sold as replacement gilts. Instead, 30-kg pigs were sold for fattening purposes after the third negative sampling result in the nursery unit. Any remaining pigs were moved to the grower rooms where they were sampled by pen on three occasions. If these pigs tested negative on all three sampling occasions, they were sent to slaughter.

In August 2021, after 11 months of repeated faecal and tissue sampling in the herd, including nine months with negative results, all restrictions were lifted, and the herd was reinstated as a gilt-producing herd. During the first six months after the restrictions were lifted, faecal samples were collected from each batch of weaned pigs approximately two weeks after weaning. Samples were also collected from the first groups of gilts that were to be sold as pregnant gilts after they were moved to the insemination room. All samples remained negative for *Salmonella* spp.. Faecal samples taken from sows within the compulsory *Salmonella *control program have also remained negative to date.

#### Epidemiological investigation

When *Salmonella* spp. was confirmed in the herd, an epidemiological investigation was undertaken to identify the possible source of contamination and the route of entry. Approximately 70 samples of feed and environmental samples from the silos, feed mill and pipes were collected. Environmental samples were also collected in the straw storage barns and from the peat that was used for newly weaned piglets. All samples collected from the feed and bedding material were negative.

The nucleus herd supplying the farm with gilts was investigated to determine if it was the possible source of the *Salmonella* infection. Faecal samples from the nucleus herd were collected twice, four weeks apart. Samples were collected as pooled samples from sows (10 animals per pool), weaned pigs (50 pigs per sock sample), growers (50 pigs per sock sample) and gilts in the insemination room (10 animals per pool). In total, 98 faecal pools and 46 sock samples from the nucleus herd were analysed, all with negative results.

Interviews with both the farm owner and the staff included questions about biosecurity measures on the farm such as rodent control, bird control, and risk of contact between wild boars and pigs. The foreign staff, originating from a country with endemic *S.* Choleraesuis, were interviewed about biosecurity routines concerning imported food and change of clothes. The interviews also included questions regarding feed (when purchased and from where) and transport logs from the feed mill were collected. The interviews did not indicate any deviations in biosecurity measures on the farm that may have led to the introduction of *S.* Choleraesuis.

Production records were also examined to see if the pathogen had had any effects on pig mortality prior to the detection. A numerical increase in preweaning piglet mortality prior to the finding of *S.* Choleraesuis was noted. During the first six months of 2020 the average preweaning piglet mortality was 18.4% while in the groups weaned prior to the finding of *S.* Choleraesuis, the mortality was 25.7%. No increases in mortality were noted for any other production stage, nor were there any apparent changes in any other productivity parameters examined.

Because the farm was situated in a wild boar-dense area, tissue and faecal samples from wild boar hunted near the farm were collected for analysis. *S.* Choleraesuis was detected in these samples. Based on these findings, surveillance for *Salmonella* spp. in the local wild boar population was initiated. The results indicated that *S.* Choleraesuis was widespread in the wild boar population in the county. Whole-genome sequencing of the *S*. Choleraesuis isolates from the herd showed that they clustered with the isolates from wild boar in the area [15].

To determine if *S.* Choleraesuis had spread from the gilt-producing herd to other herds through the purchase of replacement gilts or fattening pigs, all herds that had received pigs from the gilt multiplier in the six months prior to the first positive sampling were tested. Faecal samples were collected from all age categories of pigs from 21 herds that had purchased pigs from the gilt multiplier. In one herd that had purchased replacement gilts three weeks prior to the first positive sampling in the gilt-producing herd, two pooled faecal samples collected in the farm’s gilt quarantine unit were positive for *S.* Choleraesuis. All gilts in the unit were subsequently culled. All other samples collected from this herd were negative for *Salmonella* spp. and none of the other herds that had purchased animals from the affected herd tested positive for *Salmonella* spp. In all herds that had purchased replacement gilts from the gilt multiplier (n = 15), surveillance was continued through monthly faecal samplings until the last group of gilts purchased from the gilt producer had farrowed, or a minimum of twice with a four-week interval if the last group of purchased gilts had already farrowed. Tissue specimens from lung, liver and spleen were also collected from pigs that died with clinical signs indicative of septicaemia. In total, 4438 faecal samples and 100 tissue samples were taken during the surveillance period from these 15 herds. None of these samples tested positive for *Salmonella* spp.

## Discussion and conclusions

Until this finding, *S.* Choleraesuis had not been detected in domestic pigs in Sweden since 1979 [9]. Unlike other *Salmonella* serovars that only rarely cause clinical disease in pigs, *S.* Choleraesuis may produce severe clinical disease and high mortality in infected herds [1]. Further, *S.* Choleraesuis infection in people may also result in severe disease and death [2]. It was therefore considered to be of outmost importance to attempt to eliminate the pathogen from the affected herd and prevent it from spreading further within the domestic pig population. The repeated *Salmonella* testing done in the herd suggests that the measures undertaken to manage the outbreak were successful in eliminating the pathogen. Sampling of not only faeces but also tissues was undertaken as studies have shown that *S.* Choleraesuis may be detected in tissues such as tonsil, mesenteric lymph node and colon from infected animals that are not actively shedding the bacteria in their faeces [16, 17]. The fact that replacement gilts introduced to the herd after the outbreak remained serologically negative for 5 months post-introduction also suggests that *Salmonella* was no longer circulating in the herd.

It has been shown that most pigs naturally exposed to *S*. Choleraesuis are able to clear the pathogen between 9 and 12 weeks post infection [18] and thus, if spread from animal to animal and spread via fomites can be prevented, elimination of the pathogen should be possible. Others have reported the successful elimination of *Salmonella* spp. [19–21] from pig herds. However, these cases all relied on moving weaner or finisher pigs off-site to *Salmonella*-free facilities. In this case, the elimination was accomplished through strategic culling of *Salmonella*-shedding animals and enhancing internal biosecurity and hygienic measures in the herd. The very low number of samples that tested positive in this case may indicate that the pigs were exposed to a low dose of the pathogen [17] or that the pathogen was identified soon after it was introduced and before it had a chance to spread widely in the herd. This likely contributed to the success of the elimination plan.

*S.* Choleraesuis is rarely found in feed or animals other than pigs, and the pathogen is thought to spread mainly by horizontal transmission [22]. The only pigs introduced to this farm were replacement gilts from an approved supplier that is also included in the Swedish *Salmonella* control program. This nucleus herd was rigorously tested for *Salmonella* spp. in faeces, and because all results were negative, it was not considered a likely source of introduction. A possible source described in a Danish outbreak of *S.* Choleraesuis in 2012–2013 was direct-to-farm imported feed from areas abroad with endemic *S.* Choleraesuis [23]. This herd did not import feed from abroad and all feed stuffs, except the home-grown and purchased grain, were heat-treated. All samples collected from the feed and the storage facilities were negative and therefore feed was considered an unlikely source of introduction, although the non-heat-treated, home-grown grain could not be completely eliminated as a potential source (see below).

A possible source of introduction may have been through indirect transmission from wild boars. The pathogen was found in wild boars in the area around the farm and wild boars were often observed in the fields. Genetic sequencing of several *S.* Choleraesuis isolates from wild boar sampled in the same county as the farm have subsequently been shown to cluster with the isolate from the herd [15], which supports this hypothesis. Outbreaks of *S.* Choleraesuis have been reported in wild boars in other countries in the EU [24–26] and previous investigations have suggested that wild boar may serve as a reservoir for *S.* Choleraesuis transmission to domestic pigs [23]. Studies have shown that *S*. Choleraesuis can be recovered from dry pig faeces for at least 13 months and may remain viable and infectious for at least four months [16]. It is therefore possible that the bacteria were introduced into the herd via fomites contaminated with wild boar faeces. For example, large amounts of straw are used in Swedish pig production as enrichment and bedding material. Commonly, the machines used in the field are also used in the barn for cleaning out the straw bedding, thus providing a possible route of infection. Also, depending on wild boar density and harvesting method, the straw itself could be contaminated with faecal material from wild boars and therefore pose a risk. Similarly, the grain harvested on the farm and used in the ration without heat-treatment could also have served as a route of transmission if contaminated by wild boar faeces, although all samples from the feed and feeding system were *Salmonella*-negative.

It could be argued that the pathogen was transmitted from the infected pig farm to wild boar in the area. However, this scenario can most probably be ruled out since *S*. Choleraesuis was not only found in wild boar near the farm, but also in wild boar throughout the county. The local findings in wild boar initiated a national, ongoing surveillance program for *Salmonella* spp. in wild boars, carried out by the Swedish National Veterinary Institute and financed by the Swedish Board of Agriculture. Through this program, *S*. Choleraesuis has so far been identified in wild boars from five different Swedish counties [15]. Comparison of the genetic sequences of the isolates from the different areas has shown that they are relatively homogeneous and have a high degree of similarity to wild boar isolates from Central Europe [15]. This suggests that the outbreak in the gilt multiplier was more likely to be the result of spill over from the wild boar population rather than vice versa.

Wild boar became extinct in Sweden in the 18th century. However, after several captive wild boar kept for hunting and meat purposes escaped their enclosures in the 1970s, a wild boar population was re-established which, in recent years, has grown to over 300 000 animals [15, 27]. This relatively new and large population of wild boars in Sweden, whose range overlaps with the areas with the highest domestic pig density in the country [15], poses a risk for *Salmonella* introduction to pig herds. Indeed, since this initial outbreak, there have been three additional confirmed cases of *S.* Choleraesuis in domestic pigs in Sweden. One herd was identified through routine monitoring of pigs at slaughter. The second herd had purchased piglets from this herd and was found positive through contact tracing and the third herd was identified through traceback from a human case of *S*. Choleraesuis. These three herds had no known direct or indirect connections to the case herd described here, other than being located in the same county which has a large wild boar population that is known to carry *S*. Choleraesuis [28]. These new cases highlight the need for a better understanding of the possible transmission routes for S. Choleraesuis, including the risks that straw and non-heat-treated grains pose to the introduction of *S.* Choleraesuis into a herds located in wild-boar dense areas.

The consequences of *Salmonella* introduction in Swedish herds include not only the possibility of severe disease outbreaks but also high costs for individual herds as well as the Swedish state due to the strict legislation on the handling of *Salmonella*-positive herds. In Sweden, farmers are compensated by the Board of Agriculture for production losses, extra workload, cleaning and disinfection costs, and culled animals for 18 months after sanctions are implemented in a herd. The Board of Agriculture also covers the costs for an appointed veterinarian and diagnostic testing [10]. In total, the Board of agriculture covers 60–70% of the costs, depending on the biosecurity-level in the herd. In most cases, the farmer also carries insurance that will cover up to 80–90% of the remaining costs. In this case, approximately 90% of all costs and losses to the farmer were covered by the combination of state compensation and insurance. The cost to Board of Agriculture was SEK 7 992 234 (approx. € 689 368) to compensate the farmer for production losses, culled animals and additional costs associated with the eradication. An exact cost for the diagnostic testing in the herd is not available, but it was estimated to have been at least SEK 1 650 000 (approx. € 142 000). In this case, a test-and-cull strategy was chosen since there was a low number of positive faecal samples in the initial testing in the herd, which suggested a low level of infection in the herd. Another important factor in the choice of eradication strategy in this case was the limited access to pure-bred gilts in Sweden at the time. If a complete depopulation-repopulation strategy had been used, it would not have been possible to rebuild the whole herd and have it running at full productivity within the 18 month compensation period, which would have resulted in greater economic losses for the farmer. Thus, a depopulation-repopulation was not considered for this herd. The test-and-remove strategy, coupled with stringent hygiene measures, was successful in eliminating *S.* Choleraesuis from this herd and as such the method could be considered for use in other herds, particularly those in which *Salmonella* prevalence is low.

An interesting aspect in this case was the lack of obvious clinical signs of *S.* Choleraesuis infection. In case reports from other outbreaks of *S*. Choleraesuis, very high mortality and clinical signs including diarrhoea, respiratory problems and septicaemia among growing pigs were reported in the affected herds [7, 8]. Weaned piglets have been described by others as showing lethargy, anorexia, pyrexia, and respiratory distress [29]. According to the literature, pigs infected with *S.* Choleraesuis typically show clinical signs 36–48 h post-infection [1, 18]. In this outbreak, no increase in mortality or clinical signs among weaned pigs was recorded. However, there was a notable increase in pre-weaning mortality prior to the detection of the pathogen. At the time, outdoor temperatures were extremely high, and it was thought that this was the cause of the increase in mortality. Whether or not the introduction of *S.* Choleraesuis contributed to the increase in pre-weaning mortality could not be determined. Co-infections have previously been shown to increase the severity of *S.* Choleraesuis infection in infected herds [8, 30]. In this case, the infected herd had a high health status and was free from many common swine diseases, including PRRSV, which may help to explain the lack of clinical signs seen in this herd. It is also possible that the particular strain of *S.* Choleraesuis infecting this case herd produced milder clinical signs than those strains circulating in other herds that have reported outbreaks previously.

## Data Availability

Not applicable.

## List of Abbreviations

DDGS: Dried distillers’ grains with solubles
EN ISO: International Organizations for Standardisation
MIFA: Multiplexed Fluorescent ImmunoAssay
PCV2: Porcine Circovirus Type 2
PRRSV: Porcine Reproductive and Respiratory syndrome virus
TN70: Topigs Norsvin 70
